# The Indian Pharmacopoeia Commission: Challenge, Compliance of Pharmaceutical Industries

**DOI:** 10.34172/apb.43313

**Published:** 2024-10-03

**Authors:** Ritu Tiwari, Gaurav Sanjay Mahalpure, Poornima Gulati

**Affiliations:** ^1^Indian Pharmacopoeia Commission, Ministry of Health and Family Welfare, Government of India, Sec- 23, Raj Nagar, Ghaziabad-201002, Uttar Pradesh, India.

## Dear Editor,

 Ensuring that the pharmaceuticals available in the nation are safe, effective, and meet specified quality standards is one of the Central Government’s primary interventions to accomplish its goals in the field of public health. Drugs, cosmetics, and registered medical devices are subject to national regulatory oversight by the Central Drugs Standard Control Organization (CDSCO). Its primary focus is to ensure patient safety, rights, and well-being. It is responsible for approving drugs and clinical trials, setting standards for drugs and controlling the quality of imported drugs.^1^ Additionally, the Indian Pharmacopoeia Commission (IPC) also plays a significant role by ensuring the timely publication of the Indian Pharmacopoeia, the official compendium of standards for the identity, purity, and strength of drugs. The IPC publishes the Indian Pharmacopoeia (IP) following the Drugs Rules 1940 under it on behalf of the Ministry of Health & Family Welfare, Government of India. IPC also provides stakeholders with training on pharmacopoeial issues.^2^

## Composition of IPC

- Governing Body
- General Body
- Scientific Body
- Expert Committees and Working Groups

**Figure 1:**
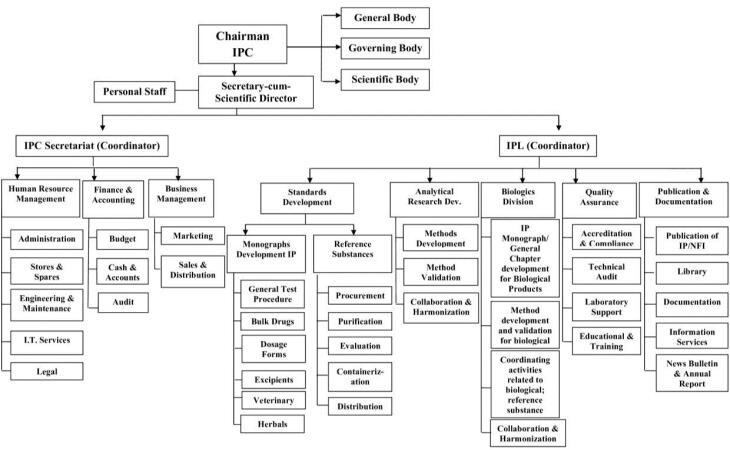
Organisational chart of Indian Pharmacopoeia Commission

Commitment to upholding the highest standards of drugs for use in humans and animals, considering the practical limits of available technologies for manufacture and analysis, is critical. By prioritizing essential medicines, aligning with international standards, and educating individuals about these standards, IPC underlines the importance of adhering to stringent quality standards in the pharmaceutical industry. This commitment is not just a goal but a necessity for our patients’ well-being and our industry’s credibility. Collaboration to monitor and report the adverse effects of pharmaceutical products and strive to become a vital resource for the Pharmacovigilance Programme of India (PvPI) is also a key objective of the IPC. The organization chart of IPC is depicted in Figure 1.

 The Indian Pharmacopoeia’s influence extends across numerous countries, demonstrating its exceptional quality and standards. It strives to standardize pharmacopoeial regulations by forming partnerships and collaborations with reputable organizations, thus facilitating the global expansion of pharmaceutical companies. It commits to advancing herbal, pharmaceutical, and medical devices and addressing challenges related to the efficacy and safety of new pharmaceuticals. The establishment of a “Herbovigilance, Phytopharmacovigilance, Pharmacovigilance, and Materiovigilance World Collaborative Centre” is an important initiative aimed at proactively monitoring and controlling adverse effects, marking a significant milestone in advancing the pharmaceutical industry.^2^ In line with its objective to elevate the standards of pharmaceutical products globally, IPC renewed its Memorandum of Understanding (MoU) with USP on November 18, 2019.^2,3^ Another MoU was signed with the British Pharmacopoeia on February 18, 2021, to exchange regulatory information on pharmaceutical products.^2,4^ It is a breakthrough for the Indian Pharmaceutical industry that the Pharmacopoeia Discussion Group (PDG) has welcomed the IPC as a participant in the global expansion pilot, which commenced at the virtual PDG Annual Meeting in October 2022.^5^ Subsequently, the MoU with USP was renewed on March 14, 2024, to sustain collaborative efforts.^2,3^ Through persistent endeavours, the Indian Pharmacopoeia has garnered recognition and acceptance in countries such as Afghanistan, Ghana, Nepal, Mauritius, Suriname, Nicaragua, Bhutan, Mozambique, Solomon Islands, and Sri Lanka under the Indian Mission, paving the way for the recognition of Indian Pharmacopoeia in countries.^2^

## Competing Interests

 All authors declare that they have no conflicts of interest.

## Ethical Approval

 Not applicable.
